# Would you like to participate in this trial? The practice of informed consent in intrapartum research in the last 30 years

**DOI:** 10.1371/journal.pone.0228063

**Published:** 2020-01-24

**Authors:** Mariana Widmer, Mercedes Bonet, Ana Pilar Betrán

**Affiliations:** Department of Reproductive Health and Research, World Health Organization, UNDP/UNFPA/UNICEF/WHO/World Bank Special Programme of Research, Development and Research Training in Human Reproduction (HRP), Geneva, Switzerland; South African Medical Research Council, SOUTH AFRICA

## Abstract

**Background:**

Informed consent is the cornerstone of the ethical conduct and protection of the rights and wellbeing of participants in clinical research. Therefore, it is important to identify the most appropriate moments for the participants to be informed and to give consent, so that they are able to make a responsible and autonomous decision. However, the optimal timing of consent in clinical research during the intrapartum period remains controversial, and currently, there is no clear guidance.

**Objective:**

We aimed to describe practices of informed consent in intrapartum care clinical research in the last three decades, as reported in uterotonics for postpartum haemorrhage prevention trials.

**Methods:**

This is a secondary analysis of the studies included in the Cochrane review entitled “Uterotonic agents for preventing postpartum haemorrhage: a network meta-analysis” published in 2018. All the reports included in the Cochrane network meta-analysis were eligible for inclusion in this analysis, except for those reported in languages other than English, French or Spanish. We extracted and synthesized data on the time each of the components of the informed consent process occurred.

**Results:**

We assessed data from 192 studies, out of 196 studies included in the Cochrane review. The majority of studies (59.9%, 115 studies) reported that women were informed about the study, without specifying the timing. When reported, most studies informed women at admission to the facility for childbirth. Most of the studies reported that consent was sought, but only 59.9% reported the timing, which in most of the cases, was at admission for childbirth. Among these, 32 studies obtained consent in the active phase of labour, 17 in the latent phase and in 10 studies the labour status was unknown. Women were consented antenatally in 6 studies and in 8 studies the consent was obtained indistinctly during antenatal care or at admission. Most of the studies did not specified who was the person who sought the informed consent.

**Conclusion:**

Practices of informed consent in trials on use of uterotonics for prevention of postpartum haemorrhage showed variability and substandard reporting. Informed consent sought at admission for childbirth was the most frequent approach implemented in these trials.

## Introduction

Quality intrapartum care is critical to the survival of women and their babies. In this sense, clinical research conducted during labour and childbirth has been crucial to improve intrapartum care and reduce maternal mortality and morbidity [1].

Informed consent is the cornerstone of the ethical conduct. By obtaining consent from participants, researchers ensure that the rights and wellbeing of the participants are protected during the clinical research. The International Confederation of Harmonization (ICH) defines the informed consent as “A process by which a subject voluntarily confirms his or her willingness to participate in a particular trial, after having been informed of all aspects of the trial that are relevant to the subject's decision to participate” [2]. It is documented by means of a written, signed, and dated informed consent form, and has three major elements: information, comprehension and voluntariness [3]. The information usually includes the research procedures, purpose, risks, anticipated benefits and a statement offering the participants the opportunity to ask questions and to withdraw at any time from the research [4–6]. Comprehension ensures that the participant has adequately understood the information [7]. Finally, the participant’s decision to participate in the research has to be voluntary, free of coercion, undue influence or intimidation [7].

In order to comply with the three elements of the informed consent, it is important to identify the most appropriate moments for the participants to be informed and to give consent, so that they are able to make a responsible and autonomous decision. However, the optimal timing of consent in clinical research during the intrapartum period remains controversial, and currently, there is no clear guidance [8–10]. While some authors believe that the informed consent should be taken during the antenatal period, others think that it is better to request the consent during labour [11, 12]. The pathway for obtaining informed consent (i.e. written vs. oral) is also the subject of discussion and analysis for optimizing and advancing research [13].

We aimed to describe specific aspects of the informed consent in intrapartum care clinical research, by assessing practices reported in studies included in the Cochrane network meta-analysis (NMA) entitled “Uterotonic agents for preventing postpartum haemorrhage: a network meta-analysis” published in 2018 [14]. As the use of uterotonics during the third stage of labour is recommended by the World Health Organization (WHO) for all women, regardless the risk of postpartum haemorrhage (PPH) or any other women’s characteristics, the Cochrane NMA offers an optimal scenario to map informed consent practices in clinical research during the intrapartum period [15].

## Methods

### Information source and eligibility criteria

This is a secondary analysis of the Cochrane network meta-analysis (NMA) “Uterotonic agents for preventing postpartum haemorrhage: a network meta-analysis” published in 2018 [14]. The Cochrane NMA included randomized controlled trials that assessed the effectiveness of different uterotonics for the prevention of PPH. The review’s protocol, methods and results are described in detail elsewhere [14]. Briefly, it included 196 randomized controlled or cluster trials of effectiveness of uterotonic agents at birth for preventing PPH following a vaginal or caesarean birth in hospital or community settings. Trials were eligible if comparisons were made between any uterotonic dosage, route of administration (oral (PO), rectal (PR), intramuscular (IM) or intravascular (IV)) or dosing regimen at birth for preventing PPH, and other uterotonic agents, placebo or no treatment. Trials evaluating uterotonic agents administered locally or not immediately after birth, or exclusively comparing different dosages, routes or regimens of the same uterotonic agent were excluded. Quasi-randomised trials and cross-over trials were also excluded.

All the reports included in the Cochrane NMA were eligible for inclusion in this analysis, except for those reported in languages other than English, French or Spanish. All full-texts were obtained.

### Data selection and data extraction

Data was extracted using a purposely designed Microsoft Excel spreadsheet. Information captured for each study included: year of publication, authors, publication title and type of report, country, sample size, participants’ characteristics (maternal age, type of pregnancy, gestational age, morbidities and pregnancy complications, as described by the authors), expected or actual mode of birth, mode of administration of the uterotonics (PO, PR, IM, IV), whether ethics approval was obtained for the study, whether information about the research was provided to participating women and when, whether consent was obtained from the women, when the consent was sought, and role in the study of the person who sought consent. Data was extracted by two reviewers independently and in duplicate. Disagreements were discussed and resolved through consensus. If needed, the third reviewer was consulted.

### Data analysis

We described the characteristics of the included studies and their populations. We classified women as low or high risk. Participants were considered low risk as classified in the primary studies or if no risk factors for themselves or the babies were listed. The year of publication of the first Consolidated Standards of Reporting Trials (CONSORT) statement was used to classify studies in two groups, before and after 1997 [16]. Countries where studies were conducted were classified in regions or in a category “more than one region involved”.

Studies were grouped on the basis of time of provision of information and time when the consent was sought, using the following categories and subcategories. Phases of labour were considered as defined by the authors:

1. Not mentioned
2. Mentioned but time not specified
3. Mentioned, and time specified:
  - During antenatal care (ANC)
  - At admission to the facility for childbirth
    - Admitted in the latent phase of labour
    - Admitted in the active phase of labour. For the purpose of this analysis this category included women in active labour or women being admitted to the labour ward
  - Labour status unknown at admission to the facility for childbirth
  - During third stage of labour
  - Either during ANC or at admission to the facility for childbirth
  - Both, during ANC and at admission to the facility for childbirth

Risk of bias of the reports included in the Cochrane NMA was conducted in the original publication [14].

## Results

**Fig 1:**
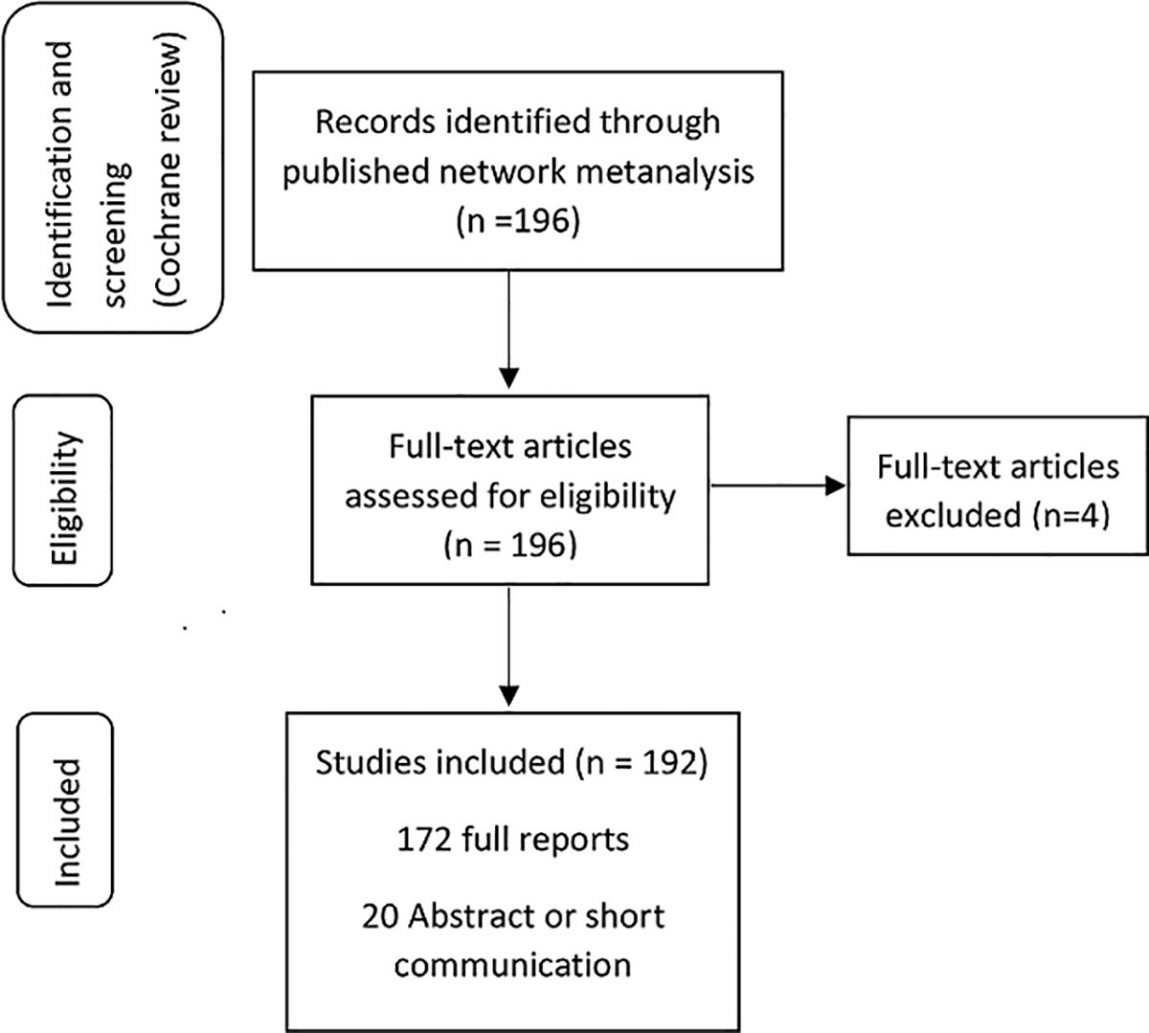
PRISMA flowchart.

A total of 192 studies were included in our analysis reporting data for 133,793 women (min 25 –max 29645 women) (Fig 1).

Four studies were excluded because they were reported in Arabic, Chinese and Thai. Table 1 shows the characteristics of the included studies and population.

**Table 1:** Characteristics of studies included in the Cochrane network meta-analysis “Uterotonic agents for preventing postpartum haemorrhage: A network meta-analysis” (2018) [14].

Study Characteristic	Number of studies	%	Number of women	%
**Total**	192	100	133,793	100
**Sample size**				
Less than 200 women	71	37.0	7,579	5.7
200–500	71	37.0	20,809	15.6
More than 500	49	25.5	105,405	78.8
Not specified	1	0.5	-	-
**Region**				
Africa	53	27.6	26,309	19.7
Asia	78	40.6	31,277	23.4
Europe	29	15.1	15,611	11.7
Latin America and the Caribbean	10	5.2	2,707	2
Northern America	11	5.7	3,563	2.7
Oceania	2	1.5	3,611	2.7
More than one region involved	4	1.5	79,796	37.2
Not specified	5	2.6	919	0.7
**Year of publication**				
Before 1997	18	9.4	11,782	8.8
1997–2001	25	13.0	31,933	23.9
2002–2009	61	31.8	29,315	21.9
2010–2018	88	45.8	60,763	45.4
**Characteristics of women**				
Low risk	134	69.8	92,040	68.8
High risk	21	10.9	3,448	2.6
Mixed low- and high-risk	25	13.0	34,051	25.5
Not specified	12	6.2	4,254	3.2
**Mode of birth considered for inclusion in the study**				
Vaginal birth	126	65.6	117,168	87.6
Caesarean section	51	26.6	9,215	6.9
Vaginal or caesarean section	3	1.6	709	0.5
Not specified	12	6.2	6,701	5.0
Mode of administration of the uterotonics*				
Oral /Rectal	18	9.4	10,487	7.8
Intramuscular	86	44.8	91,864	68.7
Intravenous	84	43.8	29,648	22.2
Not specified	4	2.1	17,94	1.3

*More invasive mode considered in the order presented in the table

The majority of studies were conducted in Asia (40.6%; 78 studies; 31,277 women) and Africa (27.6%; 53 studies; 26,309 women) although four multi-country studies contributed with 36.5% (79,796 women) of all women in the analysis. Most of the studies started in 1997 or after (62.5%; 120 studies, 101,481 women), while 6.8% were conducted before 1997, and 28.1% (54 studies; 16,322 women) did not specify the year of start. A quarter of the studies (n = 49) had sample sizes of more than 500 women but represented up to 78.8% (105,405 women) of the number of women included in this analysis. About two-thirds of the studies included only women who delivered vaginally (87.6%; 126 studies;; 117,168 women) or were low-risk (68.8%; 134 studies; 92,040 women).

Table 2 summarizes the time the information about the research was provided and the time the consent was sought.

**Table 2:** Time of provision of information and of seeking informed consent in the included studies.

	Information provided to women		Consent sought	
	Number of studies n (%)	Number of women n (%)	Number of studies n (%)	Number of women n (%)
**Not mentioned**	39 (20.3)	11,934 (8.9)	39 (20.3)	11,934 (8.9)
**Mentioned but time not specified**	115 (59.9)	71,663 (53.6)	77 (40.1)	25,400 (19.0)
**Mentioned and time specified**	38 (19.8)	50,196 (37.5)	76 (39.6)	96,459 (72.1)
**During antenatal care**	12 (6.3)	10,957 (8.2)	6 (3.1)	5,688 (4.3)
**At admission for childbirth**	15 (7.8)	27,280 (20.4)	59 (30.7)	78,889 (59.0)
**1st stage- Latent phase**	2 (1.0)	478 (0.4)	17 (8.8)	34,504 (25.8)
**1st stage—Active phase**	3 (1.6)	18,700 (14.0)	32 (16.7)	38,002 (28.4)
**Labour status unknown**	10 (5.2)	8,102 (6.0)	10 (5.2)	6,393 (4.8)
**During 3rd stage of labour**	0 (0)	0 (0)	1 (0.5)	56 (0)
**Antenatal care or at admission for childbirth**	9 (4.7)	9,657 (7.2)	8 (4.2)	9,876 (7.4)
**Antenatal care and at admission for childbirth**	2 (1.0)	2,302 (1.7)	2 (1.0)	1,940 (1.4)

About 20% of the studies did not mention whether information was provided to the participants or consent was obtained (39 studies; 11,934 women). The majority of studies reported that women were informed (59.9%; 115 studies; 71,663 women) but did not report on specific timing. When reported, most studies informed women at admission to the facility for childbirth (39.5%; 15 studies; 27,280 women).

Regarding the time of request of consent, 40.1% of the studies (77 studies; 25,400 women) did not report on specific timing. When reported, most of the studies obtained the consent once the women were admitted to the facility for birth (30.7%, 59 studies; 78,899 women). Among these, 32 studies obtained consent in the active phase of labour, 17 in the latent phase and, in 10 studies, the labour status was unknown. Women were consented only antenatally in six studies and indistinctly during antenatal care or at admission in eight studies. Findings were similar across world regions, being admission to the facility for birth the most common moment for obtaining informed consent (approximately, 30% of the studies in Africa and America and 45% of the studies in Asia and Europe).

Almost 70% of the studies (n = 129) did not specify the role in the study of the person who sought the informed consent. While 9.9% of the studies reported that the consent was obtained by the clinical staff (nurses, midwives, doctors), only 2.6% reported having a member of the research team taking this responsibility (Table 3).

**Table 3:** Practices of informed consent and reporting in included studies.

	Number of studies n (%)	Number of women n (%)
**Role in the study of the person seeking informed consent**		
Health care provider: Clinical staff (nurses, midwives, doctors), Field worker	19 (9.9%)	17,809 (13.3%)
Research team	5 (2.6%)	1,416 (1.1%)
Not specified	129 (67.2%)	102,634 (76.7%)
Not applicable	39 (20.3%)	11,934 (8.9%)
**Form of informed consent**		
Written	98 (51.0%)	101,303 (75.7%)
Verbal	3 (1.6%)	3,955 (3.0%)
Not specified	52 (27.1%)	16,601 (12.4%)
Not applicable	39 (20.3%)	11,934 (8.9%)
**Reporting of informed consent process in the study**		
Yes	153 (79.7%)	121,859 (91.1%)
No	39 (20.3%)	11,934 (8.9%)
**Approval from ethics committee mentioned**		
Yes	141 (73.4%)	119,399 (89.2%)
No	51 (26.6%)	14,394 (10.8%)

Not applicable: informed consent process was not mentioned

The informed consent pathway was not always reported. Among the 153 studies that reported conducting informed consent; 52 studies (34%, 15,890 women) did not specify the pathway, 98 studies (64%, 101,303 women) had taken written consent, and three studies (2%, 3955 women) requested verbal consent. Among these three studies, one was conducted in Europe before 1990, and the other two were conducted in Africa before 2010. (Table 3)

## Discussion

The practice of informed consent has not been uniform in studies testing uterotonics for PPH prevention conducted in the previous three decades. The timing of the provision of information and the timing of request of the consent have been sub-optimally reported. Our results suggest that, for researchers and ethics committees, the time of admission for childbirth may be the most feasible and/or acceptable timing for consent procedures in intrapartum clinical research.

To our knowledge, this is the first review to assess in a systematic and comprehensive way the practices related to informed consent process in intrapartum care research related to prevention of complications during labour and childbirth. Our results suggest that obtaining written informed consent at admission for childbirth is a common practice irrespective of the world region where the research was conducted, and this is in line with what has already been described by other authors [10, 17–19]. A previous review on research for management −as opposed to prevention− of emergency intrapartum complications showed that informed consent was mostly taken closer to the time the complication developed [19]. However, the participants eligible for a prevention trial differs from those in a treatment trial in that in the former group, women are not under the stress of a life-threatening complication. Thus, in a prevention trial, all women admitted for childbirth are potentially eligible for inclusion and not only a subgroup of women −those developing the complication− as it happens in treatment trials.

It is the duty of any researcher to guarantee the autonomy and protect the integrity of the participants during a research study. A balance is necessary so that in trying to safeguard their wellbeing, informed consent procedures do not prevent research during the intrapartum period [10]. One way to ensure that a woman makes an informed, responsible and autonomous decision in intrapartum research studies, is by creating an appropriate environment and identifying the right time to request the consent [17]. Since women may feel relatively comfortable during the latent phase of labour, researchers may consider explaining and discussing the research study to them during intervals between contractions [15, 19]. In addition, this would give the possibility to all pregnant women admitted for birth to participate in the research study, thus respecting the ethical principle of justice.

However, there are other authors who have expressed concerns about seeking informed consent during labour, mainly because women may be too stressed to understand the information they are given or because they may not have enough time to think about the research proposal and discuss it with their family or community, or pregnant women may fear being treated poorly if they refuse participation, and thus, feel compelled to accept, violating the principle of autonomy [19]^,^[11]. This risk of coercion can be reduced by separating the roles of researchers and physicians, with physicians acting as advocates for their patients [10]. In the studies included in our review such distinction was not clearly reported.

There are also ethical concerns about seeking informed consent antenatally. An important point against this option is the situation found in many low-resource settings, where large proportions of women do not attend antenatal care visits regularly, and it is therefore, very difficult to provide information about a research project prior to labour and childbirth [20, 21]. These unbooked women remain a priority for research if we aim to close the gap and the large inequalities commonly reported in maternal health [22]. Compared to women who are routinely followed during pregnancy, those who do not received antenatal care are more likely to develop complications during childbirth. Recruiting only women who were informed during the antenatal period would deny those most in need the opportunity to participate in research, violating the ethical principle of justice [23–25].

It is also important to consider women’s opinions, views and preferences. In the literature, women’s opinions are divided between the two options described above, which underlines the complexity of the decision. Some women express their preference to have the information about the research before entering labour [11]. Others, feel overwhelmed with the amount of information they have to handle during pregnancy and are not able to remember it once they are in labour [26]. Moreover, it has been described that involving pregnant women in detailed discussions about the risks and symptoms of complications they may experience during childbirth could lead to stress, and childbirth would go from being considered a normal physiological process to being a risky event [10, 17]. Stress could cause many women to refuse the invitation to participate in a research study when they are consulted during pregnancy, accepting nevertheless when invited again in labour. This might be because the fear of what will happen and the level of concern has been reduced [9]. Therefore, obtaining informed consent during antenatal care could ensure that the principle of a woman's autonomy is met, but it would not guarantee that a woman will remember the information provided about the research at the time of receiving the intervention under investigation.

Strengths of this review includes data source. This review was based on an existing Cochrane systematic review that followed a rigorous methodology for identification of eligible trials and evidence synthesis. In this sense, our results are limited to published studies and by the quality of reporting the informed consent process. We believe the exclusion of these four studies in the non-prespecified languages would have not changed substantially the results and conclusions. We did not consider the evaluation of the quality of the content of the information provided to the woman nor the content of the consent forms, or woman satisfaction with the informed consent process.

Results of this review showed that reporting of informed consent practices is not consistent across studies. This finding raise a broader question about insufficient policies on reporting ethical aspects in clinical research [10]. Although the CONSORT guidelines have contributed to the improvement of the research studies’ reporting, the reporting of ethical considerations, including ethics review board approval, is considered by CONSORT as highly desirable but not essential [27]. This lack of guidance may have contributed to the inadequate or incomplete reporting of the informed consent process in the studies included in our review.

## Conclusion

Practices of informed consent in the last three decades of clinical research with focus on prevention of postpartum haemorrhage showed variability and substandard reporting. Our results suggest that admission for childbirth may be the most feasible and/or acceptable timing for consent procedures. The lack of formal recommendations and guidelines are barriers towards more uniform research processes for informed consent during labour and childbirth. There is a need for more clear guidance on this area and we recommend the scientific community to provide leadership on establishing a process for international recommendations and standards on how the informed consent process should be best implemented and reported during labour and childbirth.

## Supporting information

S1 File PRISMA checklist. (DOC)
